# Sodium Alginate Microneedle Arrays Mediate the Transdermal Delivery of Bovine Serum Albumin

**DOI:** 10.1371/journal.pone.0063819

**Published:** 2013-05-10

**Authors:** Yusuf K. Demir, Zafer Akan, Oya Kerimoglu

**Affiliations:** 1 Department of Pharmaceutical Technology, Marmara University Faculty of Pharmacy, Istanbul, Turkey; 2 Department of Biophysics, Celal Bayar University School of Medicine, Manisa, Turkey; RMIT University, Australia

## Abstract

**Background:**

The “poke and release” strategy for the delivery of macromolecules using polymeric microneedle (MN) is of great importance because it eliminates microneedle reuse, the risks of biohazardous sharps and cross contamination, and it requires no special disposal mechanism. The main objective of this study was the determination of the stability and delivery of bovine serum albumin (BSA) that was transported across human skin via sodium alginate (SA) microneedle arrays (MNs) and SA needle free patches using two different analytical methods.

**Methodology and Findings:**

The capability of two analytical methods, the bicinchoninic acid (BCA) assay and sodium dodecyl sulfate polyacrylamide gel electrophoresis (SDS-PAGE), to precisely detect and quantify BSA within different types of polymeric MNs was assessed. The ex vivo protein release of BSA across dermatomed human abdominal skin from 10 w/w SA MNs was compared to that from needle-free patches using Franz diffusion cells. The developed applicator was mechanically characterized using a Texture Analyzer. The patch mold and its components were fabricated using a rapid prototyping machine.

**Conclusions/Significance:**

The BCA method was able to precisely detect BSA that had been loaded into SA MNs. However, the use of SDS-PAGE as the analytical method resulted in significantly different amounts of BSA recovered from differently conditioned polymeric MNs. The permeation of BSA across dermatomed human abdominal skin by SA MNs, which were composed of 100 pyramidal needles, increased by approximately 15.4 fold compared to the permeation obtained with SA needle-free patches. The ease of use of the applicator during the release studies was also demonstrated, as was its mechanical characterization.

## Introduction

The delivery of medicinal substances across the skin has been limited because of the unique barrier property of the stratum corneum (SC) [1]; however, barrier defects in the SC layer of the skin allow the penetration of environmental microorganisms into the skin to produce immunological reactions and inflammation [2]. Imperfection of SC by puncturing it with micron-scale devices has been considered as a useful approach to enhance the permeation of macromolecules across the skin [3], which are not ideal substances for conventional transdermal patch applications [4].

The primary objective of transdermal microneedle (MN) systems is the creation of microscopic holes and the transport of molecules into the skin deeper layers. Since, skin is a unique portal for vaccine administration and contains an abundance of immunocompetent cells [5], [6], numerous application strategies have been utilized for drug/vaccine delivery through painless piercing matrix systems, microneedle arrays (MNs), across the skin. Among them one of the mostly used strategy “poke and release” [7] is used in this work.

The “poke and release” application does not allow prolonged delivery, but it facilitates bolus [8], and sustained delivery if an integrated MN system used [9]. Controlled-release kinetics can also be obtained with MNs dependent upon the formulation compositions. PLGA is a material that can degrade over the course of months [10], and according to Park et al. (2006) 80% of bovine serum albumin (BSA) was released over 5 days from PLGA matrix, where the kinetic was controlled by the degradation of the PLGA MN [11].

In MNs mediated vaccination studies, a stronger and rapid immune response received [12], and this can be accomplished with the immediate release of MNs as opposed to its controlled delivery. In addition, the long-term stability of vaccines in a controlled matrix system should be carefully analyzed due to their easily degraded structure [13].

The handicap of drug delivery using “poke and release” strategy is diffusion of drug through the pores of polymer matrix, and the drawback of this strategy might be loosing a small amount of active substance during incorporation of drug with the polymer [14]. The incorporation of BSA into a melted polylactic-co-glycolic acid (PLGA) matrix at elevated temperatures (135°C) has been shown to result in a loss of protein [11]. A similar complete degradation of BSA also occurred when BSA was mixed with a melted galactose MN matrix [15].

For many reasons, the value of the “poke and release” strategy should not be underestimated, and abovementioned obstacle can be overcome. This strategy eliminates the special disposal mechanism, reuse of MN, the risk of biohazardous sharps, and cross contamination [16]. The advantages of this system are numerous as other MN strategies possess and include reductions in cold chain storage and transport, allowing self-administration/immunization regimes, which would be preferable during pandemics. Patients would not be required to go to the hospital and would not suffer the disadvantages of conventional immunization needles, such as pain, discomfort, tissue tearing, circulatory blockage, and accidental needle sticks [17]–[20].

Dissolvable carboxymethyl cellulose (CMC) [21], polyvinylpyrrolidone (PVP) [16], and dual-layer CMC [22] polymeric MN have been used in vaccine/macromolecule delivery into the skin deeper layers. These MN allow the release of the vaccine in control of polymer dissolution kinetic, and once the skin is poked with the MN, encapsulated/incorporated vaccine will be accessible to the targeted epidermal and dermal antigen-presenting cells (APCs) of the skin [20]. Abovementioned MNs generated robust immune responses even with lower antigen doses, have been found as strong as those obtained not only with intramuscular (IM) needles [16], [22], but also alongside intradermal (ID), and subcutaneous conventional hypodermic needles [23].

Moreover, dissolvable microneedle patches have been reported to successfully deliver both small (MW<500 Da) and macro molecules (MW>500 Da) in “poke and release” approach [24], [25]. According to the literature, 83% of the incorporated theophylline, and 55% and 40% of the insulin (MW of 5.6 kDa) that had been loaded only in the needles) were delivered across dermatomed porcine skin within 24 hours using 600-µm high Gantrez® AN-139 MNs [24], [25]. These MNs also increased the permeation of theophylline by 15-fold compared to the results obtained with a needle free patch [24].

An overlooked dissolvable polymer solution, sodium alginate (SA), has been found to elicit a more effective antibody response than alum [26]. Therefore, the present work examined delivery of proteins via solid, out-of-plane SA MNs in a “poke and release” fashion through the skin. Recently, using the fabrication approach and procedure outlined for the first time in [21], we have microfabricated various polymeric MNs, which are safe, inexpensive, simple, self-applicable, and consist of dissolvable: sodium alginate (SA), hydroxypropyl cellulose high (HPC-H), hydroxypropyl cellulose medium (HPC-M), swellable: cross-linked polyvinyl alcohol (PVA)–gelatin, and biodegradable: chitosan [27] materials. Here among these polymeric MNs it is desired to uniformly insert the SA MNs into the skin by means of an applicator, and thereby deliver model protein in desired concentrations and time frames, evoke rapid results, probably without causing discomfort or pain.

An applicator developed by Mechanical Engineering Department at Bilkent University was utilized to place the microneedles on the skin, and poke the SC in order to produce uniform, reproducible 100 micro holes in the skin with a 10-by-10 MNs, and thereby enhancing the permeation profiles of the loaded protein.

The protein contents of BSA-loaded SA MNs were analyzed using the bicinchoninic acid (BCA) method. Using the theoretical amount of 10 mg per MN device, the recovery of BSA was examined at different time periods (e.g., day 0, which followed a 24-hour drying period, day 7, and day 30).

The usefulness of sodium dodecyl sulfate polyacrylamide gel electrophoresis (SDS-PAGE) for detection of BSA incorporated within abovementioned polymeric MNs were evaluated. The amounts of BSA obtained from differently conditioned MNs were compared. The absorbance values acquired using the BCA method and the band intensities received through SDS-PAGE were compared to determine the capabilities of these two different methods. The evaluation and comparison of stability of the stock BSA solution under various climate conditions was also examined.

The major aim of this study was investigation of the BSA permeation profiles obtained with conventional SA patches and the formerly microfabricated SA MNs [27], which had a height of 900 µm, a width of 250 µm, an inter-base spacing of 500 µm, and an apex diameter of 100 µm. To demonstrate the noteworthy features and superiority of the SA MNs, particularly for protein delivery applications, the BSA permeation profiles were compared between dermatomed abdominal human skin from BSA-loaded SA MNs and patches. The dissolution behavior of SA MNs was evaluated to predict their usefulness for future rapid immunization studies.

## Materials and Methods

### Ethics Statement

The individuals in this manuscript have given written informed consent (as outlined in PLOS consent form) to publish these case details. Written consents from patients were taken according to the regulations of Ethics Committee of the Institute of Health Sciences at Marmara University. The ethical committee evaluated and approved consent procedure and the study protocol on July 1, 2011 with a protocol number of 38/11, and an approval number of 9.

### Design of an Applicator for the Enhancement of Microneedle Applications

**Figure 1:**
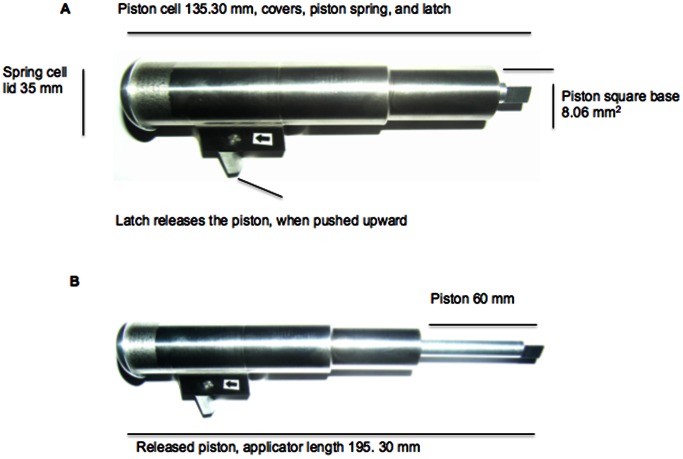
Illustration of the metal applicator used for the MN applications. (**A**) A digital photograph of the applicator prior to release of the piston. (**B**) A digital photograph of the applicator after the release of the piston. The digital images were captured using an Olympus C5060 WZ digital camera (Olympus Corporation, Lake Success, NY, USA) and were edited with Adobe® Photoshop® CS5 (Extended Version 12.0×64, Adobe Systems Inc., San Jose, CA, USA).

The applicator inspired from a published paper [28], was produced at Bilkent University. It was used to facilitate reproducible skin penetration by MNs. This metallic device was composed of a ground-ended spring, a piston, a spring cell, a spring cell lid, and latch that releases the spring upon being pressed upward. The physical dimensions of the applicator are shown in Figure 1.

Various springs supplied by a local company (Tel Cekme Sanayi, Ankara, TR) were inserted between the piston cell lid and the piston tab. Once the spring was completely attached to both sides, the lid was locked (Figure 1a). When the latch was pressed upward, the spring releases the piston toward the target (Figure 1b). The forces generated by the loaded springs in the applicator were evaluated using TA.XTPlus Texture Analyzer (Stable Micro Systems, Surrey, UK), again a modification of a published method [28], and were manipulated to cause the ground-ended springs to be squeezed in two different compression paths, short (0.1 mm) and long paths (1 mm).

### Patch and Patch Mold Manufacturing

A rapid prototyping machine (the Dimension SST 1200 es 3D Printer, Stratasys, Inc., USA) with the aid of a computer program (CatalystEX 3D Print Software for the Dimension SST 1200 es 3D Printer, Stratasys, Inc., USA) used to manufacture the centrifuge patch rack, its lid, and the patch mold in a quick and simple manner, using acrylonitrile butadiene styrene (ABS) plastic.

The ABS patch molds were constructed with a square (8.90 mm×8.90 mm) base with a depth of 3 mm and were inversely replicated for fabrication of polydimethylsiloxane (PDMS) needle-free patch molds that allow a comparison of drug delivery yielded by conventional and microneedle-based systems.

The 10 w/w SA patch and its MNs were fabricated as 10 mg of BSA in a 90-mg gel-drug formulation. These formulations were molded, and dried at ambient conditions for 24 hours and were used for the following experiments.

### BCA Method Development, Validation, and Stability of Protein Solute

A colorimetric detection method (BCA assay) was utilized to calculate amount of BSA released in vitro from the polymeric MNs and permeated BSA. Measurements of diffused BSA across dermatomed human abdominal skin and released BSA from protein-loaded polymeric MNs were calculated according to the validated BCA assay. Throughout the BCA method, guidelines of the BCA kit manufacturer were followed [29].

The absorbance of each calibration standard at a wavelength of 562 nm was determined with placing multiwell plate on microplate reader and using a spectrophotometer (BioTek Epoch, Winooski, VT, USA).

**Figure 2:**
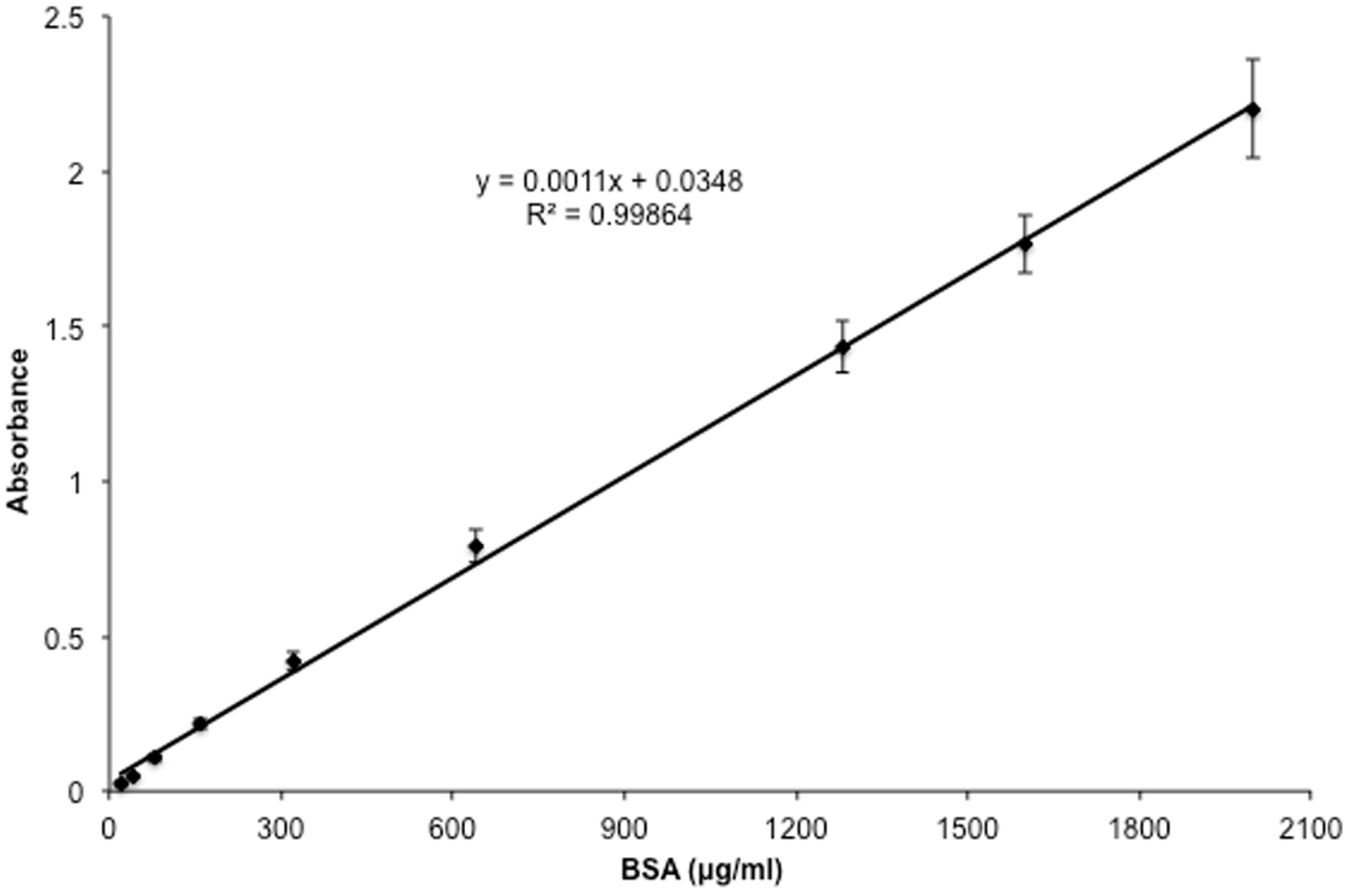
Representative mean calibration plots generated using the BCA method for determination of BSA content (means ± S.D., N = 7).

The data were analyzed using Gen5™ 2.0 Data Analysis Software (BioTek). The graphs were created in GraphPad Prism® (Version 5.0a. for Mac OS X, GraphPad Software, Inc., San Diego, CA, USA) using concentrations of standards to generate the calibration curves (Figure 2).

The linear relationships between net absorbance of the BSA standards at 562 nm and correlated concentrations were determined to generate the calibration curves (Figure 2). The limits of detection (LoD) and quantification (LoQ) were obtained as specified by the guidelines of the IUPAC [30] and ICH Topic Q2B [31].

Moreover, a comparison was performed for the stability of stock BSA solutions (1600 µg/ml in PBS, pH 7.4) that were differently conditioned, such as freshly prepared, maintained for 24 hours at 4°C (in a refrigerator), and incubated for 24 hours at 40°C and 75% RH (nominal humidity, in a climate chamber, Binder, KBF P 240, Tuttlingen, Germany).

### Analysis of the BSA/Polymeric Microneedle Interaction Using the BCA Assay

The interaction between BSA and polymeric MNs (BSA loaded with 3% w/w chitosan, 10% w/w SA, and 5% w/w HPC-H and crosslinked 20% w/v PVA with 10% w/v gelatin) was examined. The amounts of BSA recovered from freshly prepared SA MNs, as well as from SA MNs stored for 7 and 30 days were examined using the BCA assay.

The interaction between BSA and abovementioned polymeric MNs solutions was examined, similar to a paper [32], in two different cases. In both cases, the last BSA concentrations were kept constant either 1000 µg/ml or 500 µg/ml.

For these cases, 5 ml of a 2000-µg/ml and 1000-µg/ml clear BSA solutions in PBS (pH 7.4) were separately added to 5 ml of an abovementioned single polymeric MNs solution in PBS (pH 7.4). The mixtures were gently shaken for 10 minutes at 800 rpm (IKA KS125 basic shaker). The results were statistically compared to determine whether these solutions interacted.

### Analysis of the BSA/Polymeric Microneedle Interaction Using SDS-PAGE

The interactions between the protein and abovementioned polymeric MNs, as well as amounts of BSA recovered from SA MNs stored for different periods of time, were also analyzed and verified using SDS-PAGE: 4% stacking gel and 10% running gel in a mini electrophoresis cell (BioRad® Protean II, BioRad Laboratories Inc., Hercules, CA, USA). The purity and approximate molecular weight of BSA were also examined similar with a method of [33].

**Figure 3:**
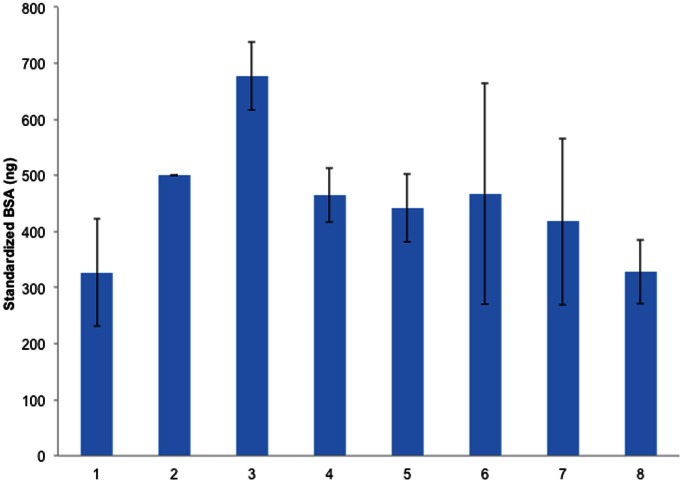
The band intensities of standardized BSA, and polymer solutions. Column 1∶250 ng standardized BSA stock solution. Column 2∶500 ng standardized BSA stock solution. Column 3∶750 ng standardized BSA stock solution. Column 4: BSA-loaded HPC MNs (500 ng of each was used). Column 5: BSA-loaded Chitosan MNs (500 ng of each was used). Column 6: BSA-loaded PVA-gelatin MNs (500 ng of each was used). Column 7: Recovered BSA from loaded sodium alginate (SA) MNs (Day 7). Column 8: Recovered BSA from loaded sodium alginate (SA) MNs (Day 30).

A clear gel containing visible bands was digitalized with a digital camera (model 500D, Canon Inc., Tokyo, Japan). After digital images were converted to gray scale mode, band intensities were measured with imaging software (ImageJ®, National Institutes of Health, USA), and recovered BSA was schemed according to the standardized band intensity (Figure 3).

### In Vitro Analysis of BSA Released from Polymeric Microneedle Arrays

A previously published method [11] was modified to study release of BSA from the polymeric MNs into solution.

To quantify amounts of BSA released from the microneedles, 10 mg of BSA was loaded into a 10 w/w SA MNs that was secured onto the bottom of rolled rim tubes composed of soda glass with PE snap-on lid vials that contained 10 ml of PBS (pH 7.4). The PBS was freshly prepared, filtered through a Millipore filtration system (Millipore Vacuum Filter System, Millipore Filter Corp., Bedford, MA, USA) using a 0.45-µm filter (Millipore Filter, 0.45 µm HVLP, Millipore Filter Corp.), and autoclaved. After the vials were filled, they were immediately incubated in a water bath shaker at 37±0.5°C, with continuous agitation at 1000 rpm (Certomat WR, B. Braun Biotech International GMBH, Melsungen, Germany). Aliquots of 200 µl of PBS were removed from each vial at various time points (5, 10, 15, 30, 60, 90, 120, 180, 240, 360, 720, and 1440 minutes) after the initiation of the experiment. An equal volume (200 µl) of autoclaved PBS (pH 7.4) at 37±0.5°C was immediately added to the vials throughout the experiment.

### Analysis of the Permeation across Human Skin of BSA from Sodium Alginate Patches and Sodium Alginate Microneedle Arrays

The passive diffusion across dermatomed human abdominal skin of the commonly used, water-soluble protein, BSA, (MW = 66 kDa) from SA (10 w/w) polymeric patches and MNs was investigated ex vivo using Franz diffusion jacketed cells with flat grounded (ground O-ring) joints composed of clear borosilicate glass with an orifice diameter of 15 mm and a receptor volume of 7 ml (Franz cell apparatus, Model 4G-01-00-15-07, PermeGear® Inc, Hellertown, PA, USA). The Franz diffusion cells were mounted on a Franz cell stirrer with acrylic cell holders, synchronously stirred using magnetic Teflon stir bars at a constant speed of 600 rpm at 220 V/50 Hz (Franz cell stirrer, model V6A, PermeGear® Inc), and thermostabilized at 37±1°C in a water bath.

Human abdominal skin flaps were collected from volunteer subjects who were scheduled to undergo abdominal reconstructive surgery at Plastic Surgery Department, Marmara University in Istanbul. After the abdominoplasty was safely performed, skin flaps of approximately 10×10 cm were removed from the patient’s body. Immediately after collection, the skin flaps were tightly mounted onto a piece of smooth wood and were trimmed to a thickness of 0.76 mm with an electric dermatome (Integra Life Sciences™, Padgett Instruments, NJ, USA) similar with a method of [34].

After the skin specimens were excised, the dermatomed samples were pre-equilibrated for 1 hour. In both the patch- and microneedles-based permeation studies, the SC side was dried with tissue paper and was secured to the vertical Franz diffusion cells with cyanoacrylate glue such with the SC and donor were facing each other, while the dermal was across from receptor.

In the patch-based permeation studies, 10 µl of ultra-pure water was exposed to the SC for initiation of adhesion. The drug-loaded patch was then immediately exposed. During this exposure, donor compartment was attached with pinch clamps to the pre-equilibrated receptor phase. All procedures of permeation study were conducted similar to a published method [24].

The SA MNs were quickly visualized with a digital microscope prior to the insertion studies. Among SA MNs that experienced even a small tip buckling were discarded, only the robust and stable ones were utilized for the drug permeation studies.

**Figure 4:**
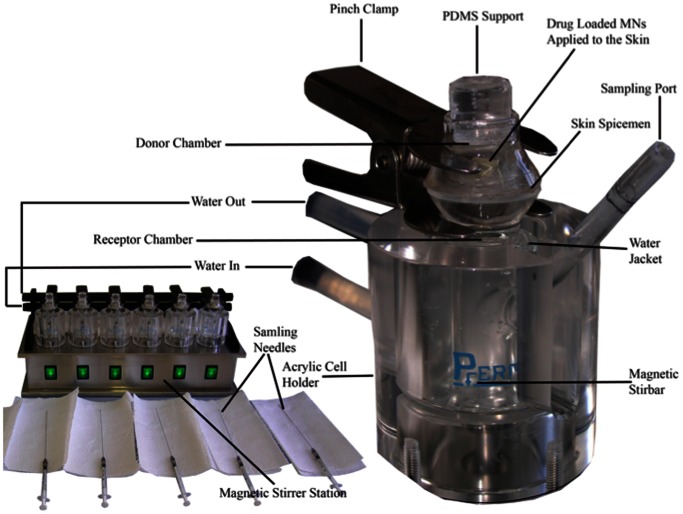
A digital image of Franz cell apparatus. Franz cell stirrer with the acrylic cell holders, the sampling needles, and the microneedle arrays (MNs)-inserted skin specimens mounted on the Franz cells (PermeGear® Inc, Hellertown, PA, USA).

In microneedle-based permeation studies (Figure 4), skin specimens were pinched with needles onto a soft blue Styrofoam support with a thickness of 1 cm. After the applicator (spring B) was applied to top of the MN base for 1 second, thereby generating a maximum force of 1.14 N, PDMS cylinder (length = 1.5 cm, cross-sectional area = 1.77 cm^2^, and weight = 0.42 g) was placed on donor chamber to serve as support material for the exposed MNs. The applicator was tailored to be inserted uniformly into the MNs and to maintain a consistent penetration throughout the diffusion studies (Figure 4).

Different cytology needles were used for each cell, and a 200-µl aliquot of receptor medium was sampled at various time points (5, 10, 15, 30, 60, 90, 120, 180, 240, 360, 720, and 1440 minutes) after initiation of the diffusion experiment. An equal volume (200 µl) of degassed fresh PBS (pH 7.4) pre-equilibrated at 37±1°C was immediately added to receptor phase with a cytology needle to maintain constant sink conditions throughout the experiment (Figure 4).

The sampled receptor medium was filtered using a Millex®-HV Syringe-Driven Filter Unit (non-sterile PVDF, Millipore Corporation, MA, USA) into HPLC vials (Agilent Technologies, Palo Alto, CA, USA) that contained the HPLC insert (250 µl, pulled point glass, Agilent Technologies, Palo Alto, CA, USA).

**Figure 5:**
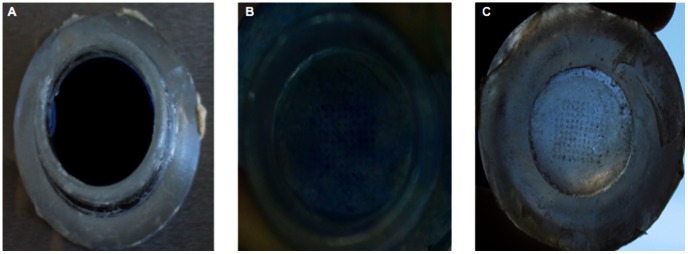
Skin micro-drilled with MNs. (**A**) A representative image of the methylene blue-filled donor compartment phase of the Franz diffusion cell, beneath which the dermatomed human skin was placed. (**B**) A representative image of the stratum corneum side of the dermatomed human skin at the end of the 24-h diffusion studies. The 10-by-10 (100) microholes that were created in the skin are shown. (**C**) A representative image of the underside of the dermatomed human skin at the end of the 24-h diffusion studies.

After the last sample was collected from the receptor phase (24 h), pinch clamps were removed, and the donor compartment was securely transferred onto tissue paper. Upper surface of epidermis was exposed with a volume of 5 ml of methylene blue solution (1000 µg/ml), where microholes had been created, for 5 minutes. The excess solution was removed, and the surface was gently washed with 10 ml of PBS (pH 7.4). The skin specimens were pictured with a digital 500D camera (Canon Inc.) [24] (Figure 5).

**Figure 6:**
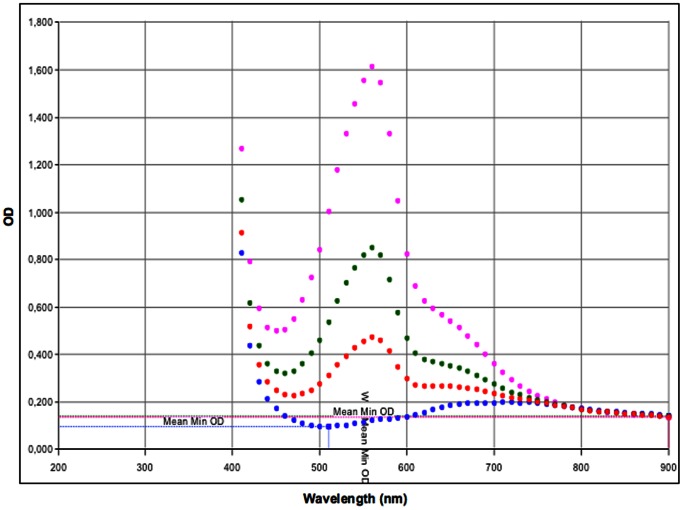
A summary of the overlapping spectral curves for permeated BSA. Spectral curves of permeated BSA through dermatomed human skin from sodium alginate (SA) patch (dark blue spots), and SA MNs (red spots) in 24 hours. Spectral curves of 0.5 mg/ml BSA solution (green spots), and 1 mg/ml BSA solution (pink spots). The measurements were obtained using the BCA kit.

**Figure 7:**
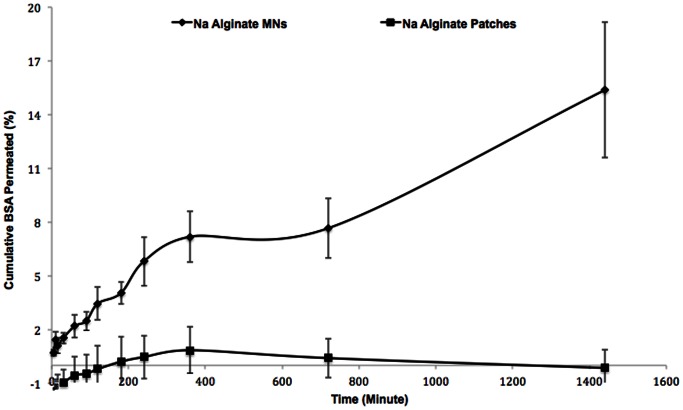
Permeation comparison of BSA (%) loaded in sodium alginate (SA) microneedles and SA patches across dermatomed human skin. Each data point represents the average of four replicates for each of 3 different polymeric microneedle arrays (N = 3).

The diffused BSA samples withdrawn from the receptor phase were evaluated using the validated BCA method (Figure 6), according to manufacturer’s protocol [29]. The percent cumulative permeated BSA was schemed as a function of time (Figure 7).

### Statistical Analysis

All of data are presented as the means ± standard deviation (SD) of replicates. The statistical analysis of effects of the different storage conditions, i.e., 4°C and 40°C/75% RH, and results of the polymer/BSA interaction experiments were performed using a two-tailed, unpaired t-test, and *p<*0.05 was considered to indicate a statistically significant difference.

The statistical comparisons of the percent BSA recovered from the SA MNs after 0, 7, and 30 days, as well as its raw absorbance, were complied with a one-way analysis of variance (ANOVA), and *p<*0.05 was considered to indicate a statistically significant difference.

The statistical comparison of the stability of BSA stock solutions that were freshly prepared, frozen for 24 hours, and incubated 24 hours was complied with a one-way analysis of variance (ANOVA), and *p<*0.05 was considered to indicate a statistically significant difference. All of the statistical calculations were conducted using GraphPad Prism® software (Version 5.0a for Mac OS X).

## Results

Table 1 shows mechanical characterization of ground-ended springs that were utilized in the developed metallic applicator to generate force. For instance, compression of springs yielded a force that was used for insertion of the MNs into the skin.

**Table 1:** Mechanical characteristic of several ground-ended springs inserted into the applicator (means ± SD, N = 5).

Spring	Internal diameterof wire (mm)	External diameterof wire (mm)	Free length(L_o_) (mm)	Number of active coils (N_a_) (mm)	Force generated with a distance of 0.1 mm (N)	Force generated with a distance of 1 mm (N)
**A**	0.60	16.33	73.65	6.0	0.15±0.02	0.14±0.01
**B**	0.80	16.50	74.91	6.0	1.05±0.01	1.14±0.00
**C**	1.00	17.07	76.16	6.4	1.34±0.02	4.03±0.01
**D**	1.20	17.48	74.60	7.0	4.98±0.00	5.95±0.01
**E**	1.20	17.82	77.06	8.0	5.21±0.09	7.68±0.01
**F**	1.50	18.25	74.65	7.1	5.61±0.08	11.90±0.04
**G**	1.50	18.67	74.80	7.2	5.73±0.04	15.40±0.04

The regression coefficient (R^2^) was 0.999 (see Table 2); therefore, the curve (Figure 2) was assumed to be linear in the concentration range (from 20 to 2000 µg/ml BSA) used for BCA colorimetric analysis. The limits of detection and quantification were examined as 23.51 and 71.26 µg/ml, respectively (Table 2).

**Table 2:** Properties of the mean calibration plot for quantification of BSA (N = 7).

Slope	y-Intercept	R^2^	RSS	LoD (µg/ml)	LoQ (µg/ml)
0.0011	0.0348	0.9986	4.76×10^−2^	23.51	71.26

Table 3 describes the interday and intraday analyses of three different concentrations (low, medium, and high) of BSA. The precision (% CV) values obtained from the interday and intraday analyses using BCA method were consistently found to be within acceptable limits (±2%).

**Table 3:** Validation of the bicinchoninic acid (BCA) method for quantification of BSA solution (means ± S.D., N = 5).

Nominal concentration (µg/ml)	Calibration equation	Mean concentrationfound (µg/ml)	S.D.	C.V. (%)	Bias (%)
**Intraday BSA**	y = 0.001034 (±0.000008345) ×+0.04214 (±0.008225)				**N = 5**
80		80.76	1.55	1.91	0.95
320		323.42	2.77	0.85	1.07
1600		1579.74	27.01	1.71	−1.27
**Interday BSA**	y = 0.001050 (±0.000005041) × +0.03237(±0.004969)				**N = 5**
80		80.67	1.60	1.99	0.83
320		321.83	2.34	0.72	0.57
1600		1575.71	23.81	1.51	−1.52

The intraday precision was found to be acceptable: the % CV values obtained with BCA method varied from 0.85 to 1.91 (Table 3). Moreover, the interday precision data were also considered acceptable: the % CV values obtained with BCA method ranged from 0.72 to 1.99 (Table 3). The bias for intraday and interday analyses was within acceptable limits (±5%); the intraday accuracy was between 98.73% and 101.07%, and interday accuracy ranged from 98.48% to 100.83% (Table 3).

The stability analysis of three different sample batches of BSA stock solution (each sample represents the mean of eight replicates) revealed that these solutions were stable for 24 hours when refrigerated at 4°C or when incubated at 40°C and 75% RH (Table 4). The % CV, bias, and recovery were 3.92%, 1.43%, and 101.43%, respectively for the samples stored at 4°C; they were 4.81%, 2.19%, and 102.19%, respectively for the samples stored at 40°C and 75% RH. A statistical comparison (an unpaired Student’s t-test) of these groups revealed that the means and variances were not significantly different; *p* was found to be 0.562 (*p>*0.05).

**Table 4:** Stability of the BSA stock solution, where the BCA method was used for BSA quantification (means ± S.D., N = 3, 8 replicates for each condition).

Stability of stock solution (1.6 mg/ml)	Assayed BSA concentration after 24 h of storage			
	Mean ± S.D.	% CV	% Bias	% Recovery
**Storage at 4°C**	1622.95±63.66	3.92	1.43	101.43
**Storage in incubator at 40°C and 75% RH**	1653.11±79.61	4.81	2.19	102.19

Table 5 shows results of BCA analysis of contents of the BSA-loaded SA MNs. The results indicated that 98.95%, 98.23% and 96.13% of pre-loaded BSA was recovered from the SA MNs after 0, 7, and 30 days, respectively (10 mg BSA was loaded onto SA MNs, which were then dried at ambient conditions for 24 hours and stored at 4°C for 7 and 30 days). A one-way ANOVA showed that there were no significant differences among theoretical BSA contents on days 0, 7, and 30 (*p>*0.05 in all cases).

**Table 5:** Assessment of the total BSA content in the Sodium Alginate (SA) microneedle arrays (MNs) after different storage periods, where the BCA method was used for BSA determination (means ± S.D., N = 3, 8 replicates of each sample).

		% BSA recovered		
		0 Day	7 Days	30 Days
**Theoretical loading of BSA (mg/array)**		10.04±0.01	10.01±0.03	10.04±0.01
**10**	mg	9.94±0.63	9.83±0.49	9.65±0.52
	%	98.95±6.20	98.23±4.81	96.13±5.20

Moreover, raw absorbance and percentage recovery values for the abovementioned MNs were also not significantly different among the BSA-loaded SA MNs that were dried for 24 hours at room temperature (without storage) and those stored at 4°C for 7 and 30 days (*p>*0.05 in all cases).

The statistical analysis of results of the polymer/BSA interaction experiments showed a significant difference between theoretical and actual BSA concentrations. These concentrations were assessed using BCA kit in presence of the polymeric MNs (See Table 6). It is conjectured that some of these polymeric MNs interfered with BCA working reagent by complexion with the Cu^2+^ ions, thereby affecting perceived BSA concentration.

**Table 6:** Analysis of the interactions of BSA with various polymeric MNs, where the BSA content was determined with BCA method (means ± SEM, N = 12).

	Types of polymeric MNs	BSA concentration in the absence of polymers (µg/ml)^a^	BSA concentration in the presence of polymers (µg/ml)^b^	*p* value	t, dF Values
**Case I**	3% Chitosan	1076.51±11.42	1017.64±15.89	0.0065*	3.008, 22
	10% SA	1097.42±14.47	1086.79±18.98	0.6606	0.4452, 22
	5% HPC-H	1046±12.38	1085.62±11.03	<0.0001**	2.37, 22
	20% PVA +10% Gel.	1046±12.38	1163.52±6.48	<0.0001**	8.39, 22
**Case II**	3% Chitosan	568.02±7.01	523.40±6.08	<0.0001**	4.81, 22
	10% SA	568.02±7.01	575.67±4.60	0.3713	0.9127, 22
	5% HPC-H	572.68±3.07	584.25±8.08	0.1945	1.34, 22
	20% PVA +10% Gel.	568.02±7.01	549.77±5.40	0.0511	2.063, 22

dF =  degrees of freedom.

a In Case I, the theoretical BSA concentration in PBS (pH 7.4) was 1 mg/ml.

a In Case II, the theoretical BSA concentration in PBS (pH 7.4) was 0.5 mg/ml.

b In Case I, both the theoretical BSA concentration and the polymeric MN concentration were 1 mg/ml.

b In Case II, both the theoretical BSA concentration and the polymeric MN concentration were 0.5 mg/ml.

*, ** Significantly different.

However, the 10% w/w SA MNs did not interact with BCA working reagent (*p*-value: 0.445) and did not affect BSA analysis. Therefore, with exception of the 10% w/w SA MNs, the MNs examined in Table 6 were not analyzed further with BCA kit for protein delivery and permeation studies. Since, BCA kit was found convenient assay for BSA loaded 10% w/w SA MNs, it was utilized in remaining experiments, and SA MNs used in protein delivery and permeation studies.

The loaded MNs were placed into SDS-PAGE wells at a hypothetical amount of 500 ng through serial dilutions of the loaded SA MNs stored at 4°C for 7 and 30 days (Figure 3 and Table 7). The SDS-PAGE analysis of BSA recovered from the SA MNs on days 7 and 30 exhibited variable band intensities, revealing that 83% and 66%, respectively of BSA had been recovered.

**Table 7:** Analysis of total BSA content in SA MNs after different storage periods, where the BSA content was determined using SDS-PAGE (means ± S.D., N = 6; the average band intensity of the fresh BSA stock was used as the standard for the determination of the BSA recovery on days 7 and 30).

		BSA recovered	
		Day 7	Day 30
**Theoretical loading** **of BSA (ng/well)**		9.99±0.01	10.04±0.01
**500**	ng	417.45±148.17	328.33±57.13
	%	83.49±29.63	65.67±11.43

A calibration plot was generated with plotting a calibration standard against band intensity. There was no sequential increase in band intensities of 250, 500, and 750 ng BSA samples in SDS-PAGE analysis of BSA content. Six different BSA concentrations ranging from 100 to 600 ng/µl were used, and they exhibited useless linearity (R^2^ value of 0.977). As a result, SDS-PAGE was not chosen for further BSA content analyses.

The results indicate that the percent cumulative release of BSA increased rapidly, even within a few minutes (attaching the donor part to the receptor part was considered as the time point zero). For instance, the percent cumulative release of BSA after 5, 10, and 15 minutes was 25.79%, 58.33%, and 92.04%, respectively. The mean concentrations of released BSA were 257.98 µg/ml, 583.43 µg/ml, and 920.70 µg/ml after 5, 10, and 15 minutes, respectively (Table 8).

**Table 8:** In vitro analysis of BSA release from 10 w/w SA MNs into solution, where the results were obtained using the BCA method (means ± SD, N = 4, four different BSA-loaded microneedle arrays were used in 4 different vials, and duplicate measurements of each vial at each time period were performed).

Time (min)	Cumulative release (µg/ml)	Cumulative release (%)
**5**	257.98±8.61	25.79±0.85
**10**	583.43±45.13	58.33±4.50
**15**	920.70±26.23	92.04±2.60
**30**	936.39±42.09	93.61±4.24
**60**	940.93±28.34	94.07±2.87
**90**	955.82±45.14	95.56±4.55
**120**	957.07±33.73	95.68±3.42
**180**	971.61±57.04	97.14±5.74
**240**	993.77±47.35	99.35±4.76
**360**	998.05±59.04	99.78±5.95
**720**	1009.45±34.61	100.92±3.50
**1440**	1021.34±42.63	102.11±4.31

The mean permeated amount and the percent of BSA permeated through dermatomed human skin after 24 hours from 10-mg BSA-loaded SA MNs were approximately 220 µg/ml and 15.4%, respectively (Figure 7, Table 9).

**Table 9:** Permeation of BSA from SA MNs across dermatomed human skin, where the results were obtained using the BCA method (means ± SD, N = 3, each data point represents the average of four replicates for each of the 3 different polymeric microneedle arrays).

Time (min)	Cumulative permeation (µg/ml)	Cumulative permeation (%)
**5**	10.03±2.20	0.70±0.15
**10**	20.81±6.47	1.45±0.45
**15**	15.89±6.19	1.11±0.43
**30**	21.90±4.41	1.53±0.31
**60**	31.52±9.23	2.20±0.65
**90**	35.57±7.54	2.49±0.53
**120**	49.49±13.10	3.46±0.91
**180**	57.80±9.08	4.04±0.63
**240**	83.22±19.60	5.82±1.37
**360**	102.80±20.30	7.19±1.42
**720**	109.7±23.80	7.67±1.67
**1440**	220.10±54.10	15.4±3.78

## Discussion and Conclusions

What impact forces will MN tips perceive when activated applicator injects the MNs? It was assumed that the applicator would cause the MNs to be inserted into skin to minimum and maximum depths of 0.1 mm and 1.0 mm, respectively, and that different forces would be produced depending on compression distance.

The mechanical measurements of forces generated in the short-distance and long-distance cases were observed. The results indicated that internal and external diameters of wire, number of coils, and free spring length were crucial parameters for generation of various impact forces as reported in a publication [28].

The impact forces generated by spring B (1.05 N and 1.14 N) were significantly smaller than forces required to axially or transversely break a single SA microneedle (0.18 N and 0.04 N, respectively). Although some of springs (C, D, E, F, and G) generated greater amounts of force than SA can withstand, and they broke microneedle arrays bases, caused tip bending, or damaged skin integrity during insertion of the needles into the skin (data not shown). The insertion of spring A into the skin was not examined [27].

The band intensities of the BSA-loaded polymeric MNs were first calculated using the calibration plot generated from SDS-PAGE results. However, the results were unreasonable, because of the R^2^ (0.977); therefore, band intensities of the BSA-loaded polymeric MNs in each gel were calculated by comparing them to band intensities of the 500-ng BSA solute (standard band). The SDS-PAGE band intensities of the BSA-loaded chitosan, HPC-H and cross-linked PVA-gelatin MNs were found to be different from those of the BSA solution alone (Figure 3).

The spectral scans of the 10 mg BSA, 10 mg BSA-loaded SA MNs, all of each were in 10 ml of PBS, produced identical OD values at 562 nm when BCA kit was used, whereas 10 ml of a blank SA MNs solution displayed an inconsiderable OD values around 562 nm, which overall was the evidence that there was not a protein-polymer interaction during quantification of BSA content, and presence of a single SA MNs does not prevent detection of BSA.

The overlapping spectral curves (data not shown), absorbance and concentration values (Table 6) of the BSA-loaded SA MNs and BSA solutions were also not significantly different at both high (10 mg/MNs) and low (5 mg/MNs) loading capacities.

The comparison of the BSA-loaded SA MNs and BSA solution produced *p* values of 0.6606 and 0.3713 for 5 mg and 10 mg BSA loadings, respectively (Table 6). As a result, BCA method was determined to be a convenient analytical method of measuring BSA within SA MNs.

An applicator with spring B was used to obtain uniform puncturing of SC side of skin samples. After the solid, out-of-plane SA MNs mediated 24-hour permeation of protein delivery, skin specimens were dyed (Figure 5a) and photographed, and a total of 100 microholes were observed on a Styrofoam support (image not shown) and the SC side (Figure 5b). Figure 5b and 5c present evidence that the SA MNs were mechanically stable, robust, and sufficiently strong to puncture the dermatomed human skin. Figure 5c illustrates that there was an absolute absence of dye leakage underneath the attached skin. Therefore, it was confirmed that upper side of the dermatomed human skin (Figure 5b) was not damaged at the end of permeation study and integrity of skin was maintained throughout permeation studies, as observed in a paper [25].

Human skin has a special ability to act as a physicochemical barrier to allergens and molecules. Drugs used in topical dermatotherapy must possess a molecular weight of less than 500 Da. This general requirement is widely known as the “500-Da rule” for transdermal drug delivery. In fact, all of known topical medications applied transdermally obey this rule [35].

This natural feature of the skin may restrict development of new pharmaceuticals if the aim is to receive a systemic drug, to allow penetration of macromolecules, or to vaccinate individuals via the transdermal route. As a crucial example, vaccines are composed of high-molecular-weight macromolecules (in the kDa to MDa range) that are not able to passively diffuse into the skin [36]. Alternative approaches have therefore been developed; for instance, usage of microneedles has been actively considered [37].

Figure 6 presents an indirect confirmation of the “500-Da rule”. As shown in the same figure, no BSA permeation through dermatomed human skin was observed at 562 nm after 24 hours when the SA patch was used as matrix cell. The spectral scans of the 0.5 mg/ml BSA solution and permeated BSA from the SA patch after 24 hours overlapped; this result demonstrates that there were significant differences between these two samples.

Exposing these MNs to cold temperatures, as well as hot and humid conditions and then analyzing them spectrally enabled analysis of stability of the BSA-loaded SA MNs. BSA recovered from freshly prepared SA MNs (day 0), as well as from MNs stored for 7 and 30 days, was also spectrally analyzed in triplicate. Spectral curves of the recovered BSA from days 0, 7, and 30 were virtually identical, which was supported by the data shown in Table 5, and shows that percent recoveries of these three groups were statistically indistinguishable.

In the present study, protein-loaded SA polymeric MNs were found to be a rapidly dissolvable microporation method. These MNs possess all of the abovementioned advantages of MNs. Therefore, to confirm their rapidly dissolvable behavior; the BSA-loaded SA MNs were examined in vitro. The release kinetics was found to behave as zero-order kinetics over a period of 24 hours. The kinetic results illustrate that these water-soluble polymeric microneedles (SA MNs) were able to dissolve within minutes and were entirely removed by the skin, thereby eliminating production of any biohazardous waste.

As opposed to a former publication [24], where alginic acid was heat treated for its gelation, and doubted that alginic acid MNs could sufficiently puncture the SC barrier [24], it is realized that its water-soluble form, sodium salt alginic acid, [38] does not require heating to form a gel. Therefore, the SA MNs were fabricated without heat treatment; finished product was firm, and strong. This work assessed the delivery of proteins across dermatomed human skin through the use of immunoadjuvant SA [26] MNs.

More importantly, the utilization of dissolvable MNs and the rapid delivery of proteins, as analyzed in this study, will no doubt generate practical, successful, short-term applications, and they are likely to be considered easy to use by patients as minimally invasive patches.

Extremely precise and accurate microfabricated pyramidal MNs [27], [39], and developed applicator enabled uniform insertion of the SA MNs into the skin without any bending, and drug permeation studies less capricious.

Figure 7 shows the permeation of BSA from the SA MNs across dermatomed human skin. As expected, the amounts of permeated BSA from the SA MNs were significantly different compared with the respective behavior in solution. For instance, 92% of the total BSA was released during first 15 minutes of the in vitro solution studies, whereas only 1% of the total BSA was permeated over the same time period during the ex vivo permeation studies across dermatomed human skin (Table 9). However, continuous rise in the delivered BSA amounts was observed throughout the permeation study.

A drawback of the “poke and release” approach is protein present in the needle itself can be delivered; therefore some protein in the base section of the microneedle array are wasted, as observed in this study. This obstacle might be overcome by incorporating the macromolecule only to the needle section of the array, and should not happen in case of usage of small molecular weight drug.

In conclusion, the results indicate that the SA patches were unable to deliver BSA, whereas the MNs delivered significant amounts of BSA across the skin within the same period of time and under the same experimental conditions. The ex vivo permeation of BSA across dermatomed human abdominal skin from an SA MN array with 100 pyramidal needles increased the permeation of this protein by approximately 15.4 fold compared to its needle-free patch.

Because of the noteworthy dissolution behavior, and impact of the SA MNs especially in protein delivery, the SA MNs could be used as dissolvable matrix for rapid immunization studies. The commercialization of these polymeric microarrays would be of great value to patients. Due to possessing great potential to revolutionize the vaccination approaches and conventional patches, utilizing these MNs as drug delivery device will no doubt create a large economic impact.
